# Vertical Soil Profiling Using a Galvanic Contact Resistivity Scanning Approach

**DOI:** 10.3390/s140713243

**Published:** 2014-07-23

**Authors:** Luan Pan, Viacheslav I. Adamchuk, Shiv Prasher, Robin Gebbers, Richard S. Taylor, Michel Dabas

**Affiliations:** 1 McGill University, 21111 Lakeshore Road, Ste-Anne-de-Bellevue, QC H9X 3V9, Canada; E-Mails: luan.pan@mail.mcgill.ca (L.P.); shiv.prasher@mcgill.ca (S.P.); 2 Leibniz-Institute for Agricultural Engineering (ATB), 100 Max-Eyth-Allee, Potsdam 14469, Germany; E-Mail: rgebbers@atb-potsdam.de; 3 DUALEM, 540 Churchill Avenue, Milton, ON L9T 3A2, Canada; E-Mail: rtaylor@dualem.com; 4 Geocarta, 5 rue de la Banque, Paris 75002, France; E-Mail: michel.dabas@geocarta.net

**Keywords:** galvanic contact resistivity, apparent soil electrical conductivity, on-the-go soil sensing

## Abstract

Proximal sensing of soil electromagnetic properties is widely used to map spatial land heterogeneity. The mapping instruments use galvanic contact, capacitive coupling or electromagnetic induction. Regardless of the type of instrument, the geometrical configuration between signal transmitting and receiving elements typically defines the shape of the depth response function. To assess vertical soil profiles, many modern instruments use multiple transmitter-receiver pairs. Alternatively, vertical electrical sounding can be used to measure changes in apparent soil electrical conductivity with depth at a specific location. This paper examines the possibility for the assessment of soil profiles using a dynamic surface galvanic contact resistivity scanning approach, with transmitting and receiving electrodes configured in an equatorial dipole-dipole array. An automated scanner system was developed and tested in agricultural fields with different soil profiles. While operating in the field, the distance between current injecting and measuring pairs of rolling electrodes was varied continuously from 40 to 190 cm. The preliminary evaluation included a comparison of scan results from 20 locations to shallow (less than 1.2 m deep) soil profiles and to a two-layer soil profile model defined using an electromagnetic induction instrument.

## 1. Introduction

Information about soil quality and its variations is relevant for agriculture, archaeology, and environmental assessment. Traditional methods for soil exploration are laborious and, thus, not economical for high-resolution soil mapping. Proximal soil sensing is a new discipline that combines soil sensors and data analysis methods to obtain high resolution soil information at a reasonable cost. Sensors for apparent electrical conductivity (*EC_a_*) and its reciprocal apparent electrical resistivity (*ER_a_*) have been the most popular for proximal soil sensing. The ability of soil to conduct and accumulate an electrical charge has been linked with several physical and chemical soil properties. Therefore, spatial changes in soil *EC_a_* have been linked to spatial soil heterogeneity [1–4]. In non-saline, non-hydromorphic mineral soils, the particle size distribution (soil texture) in combination with related soil attributes is typically the most influential factor for spatial variation of soil *EC_a_* [5], as smaller particles (clay and fine silt) are related to higher ion concentrations (greater surface charge) and superior water storage (smaller pore size).

Soil *EC_a_* (or *ER_a_*) is commonly measured by a galvanic contact, capacitive coupling or electromagnetic induction (EMI) [5,6]. In each case, an instrument is pulled across the landscape while recording periodic measurements along with their geographic coordinates. Therefore, collected data is a series of measurements representing a specific soil depth, depending on the configuration between electrical signal transmitting and receiving elements. Although such data can be adequate for a number of applications, knowing how soil *EC_a_* and related properties change with depth in different parts of a field can be equally important. For example, the depth and physical composition of topsoil along with the nature of subsoil material characterize the root development environment in relation to the mechanical impedance to root growth, soil water and nutrient storage capacity as well as the potential activity of microorganisms.

Several different methods have been used to estimate how apparent soil electrical conductivity changes with depth. *In-situ* methods, such as soil coring, are invasive and can only be performed in a stop-and-go mode. Surface methods, such as vertical electrical sounding, are non-invasive or produce negligible soil disturbance and most can collect data on-the-go [7]. The depth of investigation for surface methods is altered by changing the parameters of the instrument. For example, a depth response curve for an EMI instrument can be modified by changing the distance between transmitting and receiving coils, their orientation, height above ground, or electrical frequency [8]. Likewise, the geometrical configuration between current injecting and sensing electrodes is related to depth sensitivity when using galvanic contact resistivity systems [7].

**Figure 1.**
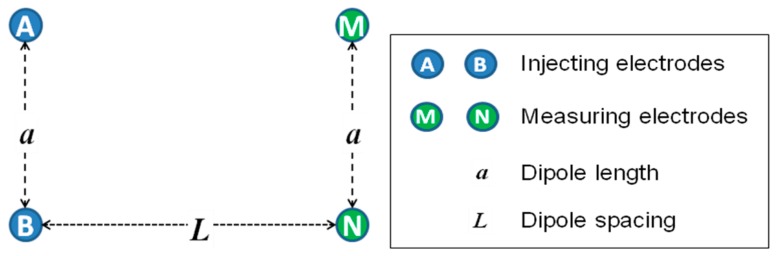
Equatorial dipole-dipole electrode configuration.

There are several common geometrical configurations used by popular galvanic contact resistivity instruments, such as Schlumberger, Wenner, and dipole-dipole arrays [7]. For example, Figure 1 illustrates the equatorial dipole-dipole array. In this case, soil electrical resistivity and conductivity are calculated using the following equations [9]:

(1) $$ER_{a}=\frac{\pi }{\frac{1}{L}-\frac{1}{\sqrt{\alpha^{2}+L^{2}}}}\cdot \frac{U}{I}$$

(2) $$EC_{a}=\frac{1000}{ER_{a}}$$

where *ER_a_* is electrical resistivity [Ω·m]; *a* is dipole length (inter-pair electrode spacing) [m]; *L* is the distance between the injecting and the measuring pairs [m]; *U* is the voltage drop along the distance *L* [mV]; *I* is the generated current [mA]; *EC_a_* is electrical conductivity [mS/m].

**Figure 2.**
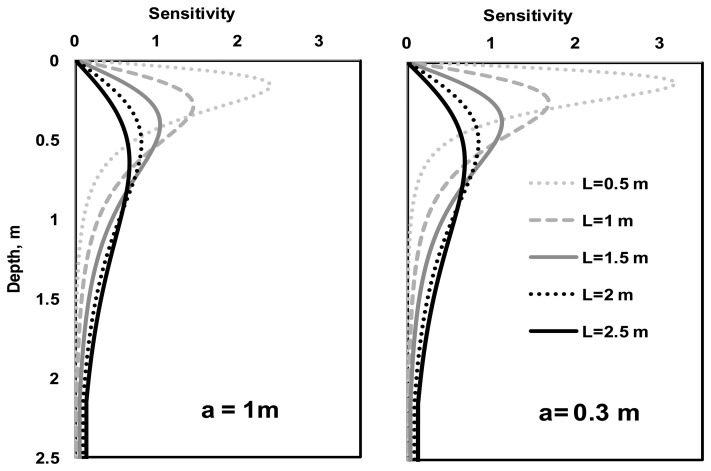
The sensitivity-depth-curves with dipole spacing (*L*) varying from 0.5 m to 2.5 m with 0.5 m increments and dipole lengths (*a*) of 1 m and 0.3 m.

Figure 2 shows several depth sensitivity curves with dipole spacing (*L*) varied from 0.5 to 2.5 m and dipole lengths (*a*) being either 1 or 0.3 m. It can be seen that the depth of investigation is a function of dipole length and spacing.

To obtain multiple *EC_a_* measurements representing different depths, some galvanic contact resistivity instruments include several pairs of measuring electrodes. For example, the Veris^®^ 3100 (Veris Technologies, Inc., Salina, KS, USA); the system has six Coulter electrodes arranged in a Wenner array with two depths of investigation. Dabas [10] developed a sensor system with four pairs of rolling electrodes, which enables three depths of investigation. Later, Lueck and Ruehlmann [11] developed another sensor system, which permitted measurements at five depth levels using twelve rolling electrodes. For these systems, each pair of electrodes at a fixed distance provides a single depth of investigation.

With an EMI instrument, it is feasible to use a pneumatic angular scanning system to measure *EC_a_* incrementally through a range of depths of investigation [12]. Similar measurements can be obtained with a galvanic system by changing the spacing between electrodes. Therefore, the objective of this study was to develop a prototype galvanic contact resistivity system capable of obtaining relatively continuous scans of *EC_a_* using two pairs of rolling electrodes configured in an equatorial dipole-dipole mode with variable distances between the pairs.

## 2. Materials and Methods

### 2.1. Scanner System Development

**Figure 3.**
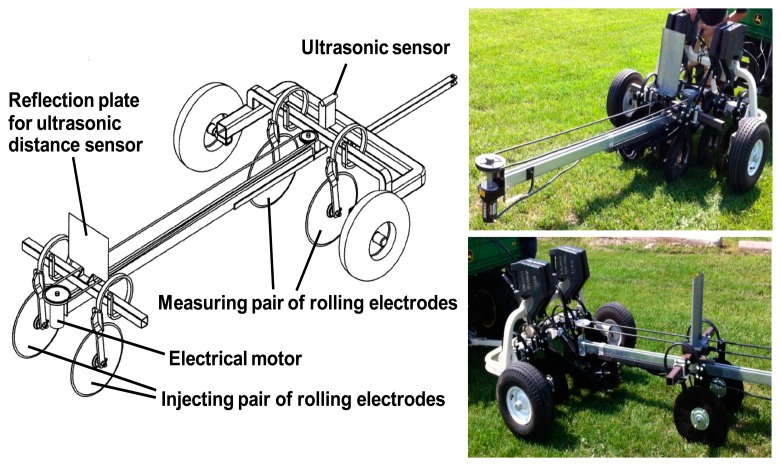
Prototype galvanic contact resistivity scanner.

Shown in Figure 3, a scanner system was developed using four Veris^®^ Q series disc electrodes (Veris Technologies, Inc.) configured as shown in Figure 1. An electrical motor automatically moves the electrodes through a range of spacing. A 4-Point light hp (Lippmann Geophysikalische Messgeräte, Schaufling, Germany) instrument was used to conduct the measurements with the current set within the range of 0.1 to 100 mA, depending on the observed *EC_a_* values. Since insufficient suppression of mains interference may occur if the mains frequency is a multiple of the measurement frequency, the frequency was set at 25 Hz to avoid this phenomenon (frequency of mains in Canada is 60 Hz). The distance between the current injecting and measuring pairs of rolling electrodes was varied continuously from 40 to 190 cm. The distance between each pair of electrodes was fixed at 45 cm. A ToughSonic TSPC-30S1-232 ultrasonic sensor (Senix^®^ Corporation, Hinesburg, VT, USA) was used to measure the distance between the two pairs of electrodes while a Global Positioning System (GPS) receiver measured the geographic coordinates. All data were recorded using a LabView (National Instruments, Inc., Austin, TX, USA) interface developed for this application. As implemented, the electrodes moved through a complete cycle of spacing in less than 10 s in either a stationary mode or on-the-go. To filter out the noise, each complete scan was converted to an array of fifteen (arbitrarily defined) discrete *EC_a_* measurements corresponding to different depth sensitivity curves. For preliminary system evaluation, these surface data were used to estimate the parameters of a two-layer model of electrical conductivity by means of inversion [8].

### 2.2. Preliminary Field Evaluation

#### 2.2.1. Test Locations and Direct Soil Profiling

**Figure 4.**
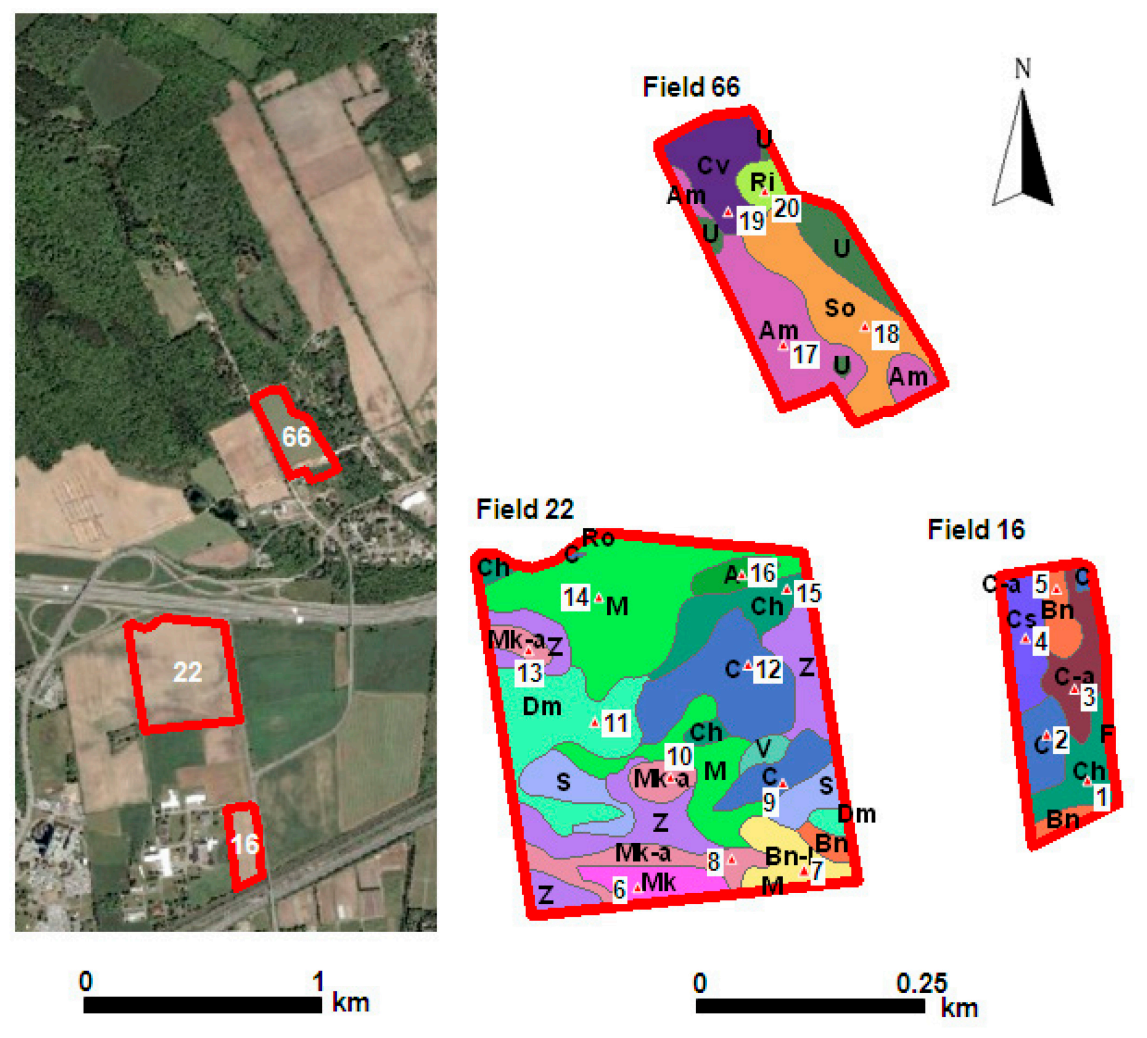
Experimental fields with different soil types. Notes: Bb, D and Ro: Clay; Am and V: Loamy sand; Ch, M and Ri: Clay Loam; Mk and Mk-a: Organic deposits; C: Fine sandy loam; So and U: Sand; Bn-1: Ill-drained loam; Cv: Sandy loam; Bt, Cs, Dm and O: Light sandy loam; C-a: Shallow find sandy loam; Bn and F: Loam; S and Z: Silt loam.

The scanner system has been tested in three agricultural fields at Macdonald Farm of McGill University, Ste-Anne-de-Bellevue, QC, Canada (Figure 4).

**Figure 5.**
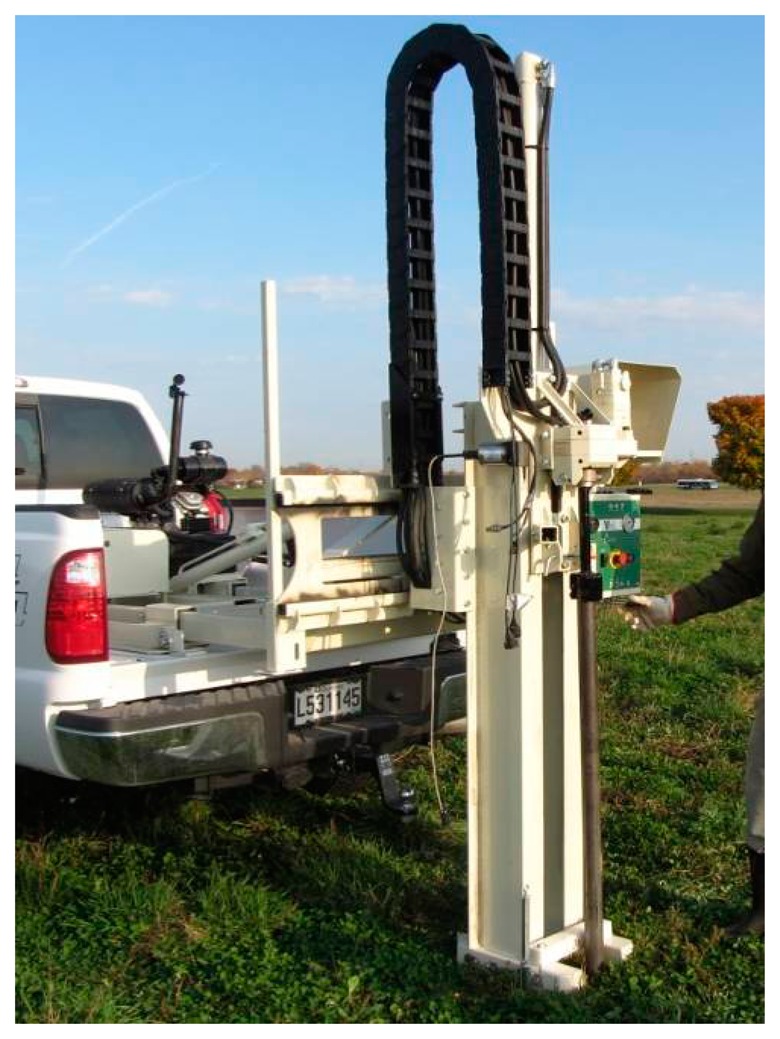
Veris^®^ P4000 unit with hyperspectral, *EC_a_* and force sensors.

Twenty sites with a wide range of soil textures were selected to evaluate the system's performance in the stationary mode. To characterize the soil profiles in each location, a Veris^®^ P4000 unit (Veris Technologies, Inc.) shown in Figure 5 was used with either the integrated soil probe or the soil core extraction attachment. The probe allowed for the collection of direct (*in-situ*) *EC_a_* measurements with 2.5–3 cm depth increments. Unfortunately, the soil profiles obtained in Fields 16 and 22 were very short due to the shallow and very hard subsoil (heavy clay or bedrock). All the cores that could be collected were divided by homogeneous depth intervals or horizons according to color and texture by feel. In the laboratory the soil texture of each horizon was more accurately determined using the hydrometer method [13]. However, no information was available for the characteristics of the subsoil. Therefore, direct validation of the *EC_a_* scanner data was not feasible and only four soil profiles from Field 66 were used to visually observe the agreement between 1-m deep soil profiles and corresponding scanner system measurements.

#### 2.2.2. Indirect Soil Profiling

**Figure 6.**
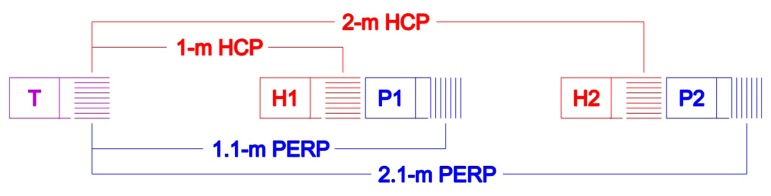
Schematic overview of the DUALEM-21S sensor with the transmitting coil (T) and four receiving coils (two in a horizontal coplanar (HCP) and two in a perpendicular (PERP) loop orientation in relationship to the transmitting coil) [18,19].

To overcome the limitation of unknown soil profiles below the penetrable topsoil layer, a DUALEM-21S (DUALEM, Milton, ON, Canada) EMI instrument with four depths of investigation [14,15] was used for the validation of the scanner system measurements (Figure 6). The DUALEM-21S measurements were interpreted according to a two-layer (three parameters) model, as more complex models would require more than four different depth sensitivity curves. The thickness of a superficial layer, the *EC_a_* of this layer and the *EC_a_* underlying earth were estimated as those that minimize the error between the predicted and the measured overall *EC_a_* values according to Equation (3). The predicted values were calculated using Equations (4–6) [16–19].

(3) $$\varepsilon =100\sqrt{\frac{{\left(H1\prime -H1\right)}^{2}+{\left(P1\prime -P1\right)}^{2}+{\left(H2\prime -H2\right)}^{2}+{\left(P2\prime -P2\right)}^{2}}{{\left(H1\right)}^{2}+{\left(P1\right)}^{2}+{\left(H2\right)}^{2}+{\left(P2\right)}^{2}}}$$

(4) $$\begin{array}{c} x\prime =EC_{1}\left(n-m\right)+EC_{2}\left(1-n\right)\\ x\prime =H1\prime ,P1\prime ,H2\prime ,\text{or}P2\prime \end{array}$$

(5) $$m=\{\begin{array}{ccc} 1-\frac{1}{\sqrt{4h^{2}+1}} & \text{for} & H1\\ \frac{2h}{1.1\sqrt{4{\left(\frac{h}{1.1}\right)}^{2}+1}} & \text{for} & P1\\ 1-\frac{1}{\sqrt{4{\left(\frac{h}{2}\right)}^{2}+1}} & \text{for} & H2\\ \frac{2h}{2.1\sqrt{4{\left(\frac{h}{2.1}\right)}^{2}+1}} & \text{for} & P2 \end{array}$$

(6) $$n=\{\begin{array}{ccc} 1-\frac{1}{\sqrt{4{\left(h+d\right)}^{2}+1}} & \text{for} & H1\\ \frac{2\left(h+d\right)}{1.1\sqrt{4{\left(\frac{h+d}{1.1}\right)}^{2}+1}} & \text{for} & P1\\ 1-\frac{1}{\sqrt{4{\left(\frac{h+d}{2}\right)}^{2}+1}} & \text{for} & H2\\ \frac{2\left(h+d\right)}{2.1\sqrt{4{\left(\frac{h+d}{2.1}\right)}^{2}+1}} & \text{for} & P2 \end{array}$$

where *h* is the height of the DUALEM-21S sensor above the ground [m]; *d* is the depth of the surficial layer [m]; *EC*_1_ is the apparent electrical conductivity of the surficial layer [mS/m]; *EC*_2_ is the apparent electrical conductivity of the underlying earth [mS/m]; *H*1, *P*1, *H*2, *P*2 are the *EC*_a_ values measured, respectively, by the four arrays of the DUALEM-21S [mS/m]; *H*1′, *P*1′, *H*2′, *P*2′ are the predicted *EC*_a_ values [mS/m]; ε is the error between predicted and measured *EC*_a_ values.

### 2.3. Soil Profile Model Parameters Prediction

Assuming that the two-layer models obtained from EMI were acceptable approximations of the changes of electrical conductivity with depth, a multilayer perception neural network algorithm [20–24] was developed to define the relationship between the scanner system measurements and the three model parameters (*d*, *EC*_1_ and *EC*_2_). This was performed using IBM-SPSS Modeler Version 15.0 (IBM-SPSS, Inc., Chicago, IL, USA) software. The scanner system data (over 200 measurements during each scan) were aggregated using fifteen discrete distances with 10-cm increment (from 40° to 190° cm). They were assigned as inputs (*X*_1_ through *X*_15_). The three model parameters *Y*_1_ = *d*, *Y*_2_ = *EC*_1_ and *Y*_3_ = *EC*_2_) were set as targets to be predicted. At this preliminary stage, all scans were treated as calibration data. Additional diverse scans would be required to make this validation procedure more complete by splitting the scans between calibration and validation datasets.

A simple linear regression was used to relate individual model parameters estimated from EMI and those predicted using the neural network from the scanner system measurements. To report results of this analysis in physical units, the prediction error (PE) for each model parameter was calculated according to:

(7) $$\text{PE}=\sqrt{\frac{1}{N}\cdot \sum_{i=1}^{N}{\left(x_{i}-x_{i}^{\prime }\right)}^{2}}$$

where *x_i_* is a soil profile model parameter estimated using EMI; $x_{i}^{\prime }$ is the corresponding soil profile model parameter predicted using the scanner system measurements processed through the neural network; *N* is the number of compared two-layer soil profile models.

## 3. Results and Discussion

**Figure 7.**
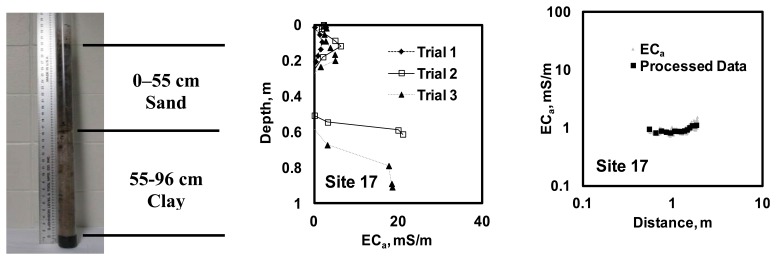

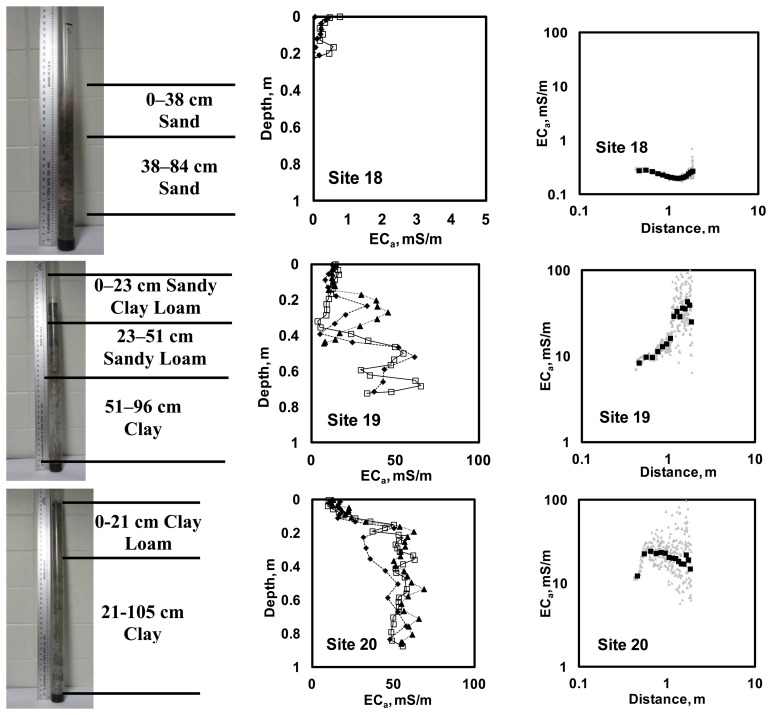
Example of soil profiles (**left**) along with the corresponding apparent soil electrical conductivity profiles measured by Veri^®^ P4000 unit (**middle**) and the scanner system measurements (**right**).

Figure 7 illustrates four sample soil profiles and *EC_a_* measurements obtained using a Veris P4000 along with the corresponding measurements obtained using the scanner system.

With no substantial difference in soil salinity (laboratory EC < 200 μS/cm) and water content (20–25 g/g gravimetric water content), the results of the particle size analysis were consistent with the *in-situ EC_a_* measurements, in that coarse soil texture produced relatively low values of *EC*_a_. Likewise, the relationship of the scanner system *EC*_a_ measurements and the distance between current injecting and sensing pairs of electrodes were consistent with these soil profiles. Thus, sandy site 18 produced a relatively constant scan with low *EC*_a_ values. Sites 19 and 20 indicated higher *EC*_a_ values (as well as their variance) with greater distances between the injecting and measuring pairs; this was consistent with an increase in clay content with depth. With limited information about soil profiles, similar observations were made when analyzing the results from the other sixteen sites.

**Figure 8.**
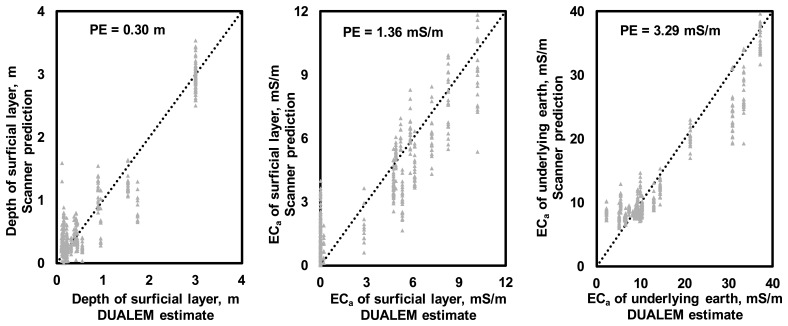
Relationships between the soil profile model parameters estimated using DUALEM-21S and those predicted using the scanner system measurements processed through the neural network while treating the entire dataset as calibration data.

**Figure 9.**
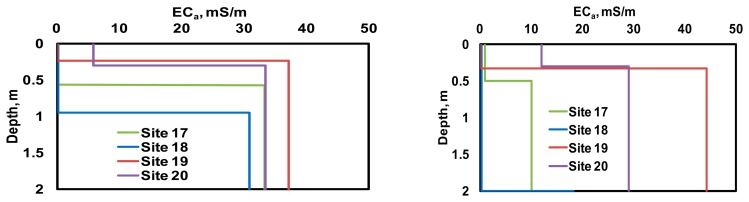
Soil profiles modeled using DUALEM-21S (**left**) and scanner system (**right**) measurements.

Figure 8 illustrates the relationships between the two-layer soil profile model parameters estimated using DUALEM-21S data and those predicted using the scanner system data. Based on these results, PE of 0.3 m could be expected for the depth of the upper soil layer. PE estimates of *EC*_a_ were 1.36 mS/m for the upper soil layer and 3.29 mS/m for the subsoil. Figure 9 illustrates the soil profile models found for the four sites illustrated in Figure 7. Again, despite the insufficient number of model parameters needed to describe the soil profiles observed through the test locations, the results were consistent with soil profiling observations.

Based on the achieved design, continuous scanning of galvanic contact soil resistivity could be applied to predict the change in soil physical properties with depth. These estimates can be spatially interpolated to obtain a 3D representation of *EC*_a_. In principle, this method resembles the accepted geophysical practices of vertical electrical sounding and 2D soil electrical resistivity tomography that are typically conducted by manually inserting electrodes using a predefined geometrical pattern [25]. However, the instrument developed is also a mobile *EC*_a_ mapping instrument that can be used to collect georeferenced measurements along a path of travel [5]. The advantage of the scanner developed for this study is the quasi-continuous variation of depth sensitivity and the lower number of electrodes when compared to the multi-electrode galvanic coupled sensors with fixed geometries. Unfortunately, the preliminary field evaluation performed in this study was limited due to the unknown soil profile below the depth of the extracted soil cores. Further work is needed to evaluate the practical applicability of the newly developed platform and to optimize the operational and data interpretation parameters.

## 4. Conclusions

An automated scanner system with an equatorial dipole-dipole array was developed and tested in several agricultural fields. At sites where soil profiling was feasible, the extracted soil cores indicated changes in soil textures with depth consistent with the direct *EC*_a_ profiles and the scanner system measurements obtained. Furthermore, a neural network calibration model could be used to predict the parameters of a two-layer soil profile model estimated using a commercial multi-depth EMI instrument, which revealed 0.3 m expected difference in predicting the depth of topsoil in a two-layer model with 1.36 and 3.29 mS/m *EC*_a_ prediction uncertainties for the superficial layer and underlying earth, respectively. However, to predict the parameters of a more complex model, it is necessary to know the soil profiles at greater depths.
